# A Case of Eltrombopag-Induced Thrombotic Microangiopathy Initiating Hemodialysis

**DOI:** 10.7759/cureus.75947

**Published:** 2024-12-18

**Authors:** Haruka Fukuda, Mineaki Kitamura, Sayaka Sugiyama, Atsushi Sawase, Hiroshi Yamashita, Hideki Tsushima, Junji Irie, Eisuke Katafuchi, Toshiyuki Nakayama, Hiroshi Mukae, Tomoya Nishino

**Affiliations:** 1 Nephrology, Nagasaki Harbor Medical Center, Nagasaki, JPN; 2 Nephrology, Nagasaki University Hospital, Nagasaki, JPN; 3 Hematology, Nagasaki Harbor Medical Center, Nagasaki, JPN; 4 Pathology, Nagasaki Harbor Medical Center, Nagasaki, JPN; 5 Pathology, University of Occupational and Environmental Health, Kitakyushu, JPN; 6 Respiratory Medicine, Nagasaki University Graduate School of Biomedical Sciences, Nagasaki, JPN

**Keywords:** antiphospholipid syndrome, eltrombopag, end-stage renal disease (esrd), idiopathic thrombocytopenic purpura, thrombotic microangiopathy

## Abstract

Thrombopoietin receptor agonists are used in addition to steroids for idiopathic thrombocytopenic purpura. A 55-year-old male with idiopathic thrombocytopenic purpura, treated with eltrombopag, developed a rapid decline in renal function following the increase in eltrombopag dose. Renal biopsy showed glomerular endothelial disorder and platelet thrombus, which suggested eltrombopag-induced renal-limited thrombotic microangiopathy. He was treated with steroids; however, his renal function did not improve, and hemodialysis was initiated. During the treatment, acute myocardial infarction and arteriovenous fistula obstruction occurred. After that, he was subsequently diagnosed with antiphospholipid antibody syndrome. Eltrombopag can cause thrombotic microangiopathy and, in some cases, result in renal failure. Patients with idiopathic thrombocytopenic purpura coexisted with antiphospholipid antibody syndrome are at increased risk of thrombosis with thrombopoietin agonists and require careful follow-up.

## Introduction

Idiopathic thrombocytopenic purpura (ITP) is an acquired disease that causes thrombocytopenia through immunological pathways such as anti-platelet antibodies. Thrombopoietin receptor agonists (TPO-RAs), which bind thrombopoietin receptors and promote differentiation and maturation of blood megakaryocytic cells, have been used as a second-line therapy for ITP. TPO-RAs could increase platelet sustainably and decrease the dose of steroids in refractory and tolerance ITP cases [1-2]. However, adverse events like thrombosis, filamentation of bone marrow, and hepatic dysfunction were reported in cases with TPO-RAs, and careful observation is required after administrating TPO-RAs. We report a case of thrombotic microangiopathy caused by eltrombopag, which required the initiation of maintenance hemodialysis.

## Case presentation

A 55-year-old man was referred to the nephrology department at our facility with symptoms of fatigue and rapid progressive renal failure. He was diagnosed with idiopathic thrombocytopenic purpura (ITP) 21 years ago and treated with prednisolone. He refused to undergo splenectomy and continued to be treated with small amounts of prednisolone. In May 2014, prednisolone was gradually reduced to 1 mg/day, and platelets remained around 50,000/μL. However, in April 2020, platelets decreased by 8,000/μL, and the prednisolone dose was increased to 10 mg/day. Eltrombopag 12.5 mg/day was initiated in March 2021 because his platelet levels declined after decreasing the prednisolone dose.

**Figure 1:**
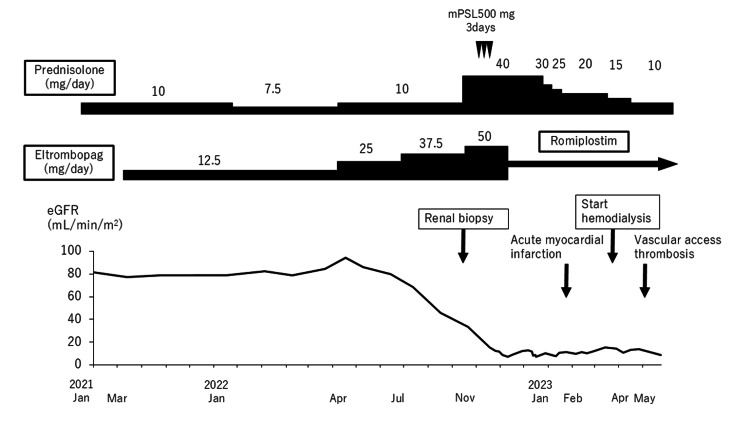
Clinical course of this case

His creatinine was around 0.8 mg/dL from 2020 to 2021; however, after increasing eltrombopag 12.5 to 25 mg/day in April 2022, his renal function declined rapidly (Figure 1). His creatinine levels increased rapidly from 1.75 mg/dL in late September, 3.47 mg/dL in late October, and 4.36 mg/dL in early November. Consequently, he was admitted to our department to conduct further investigations. The patient also had a history of shingles in April 2022. He was a former smoker but denied alcohol use in the past. He had never had a history of allergies. Additionally, there was no family history of renal or hematological disorders. His regular medications were as follows: eltrombopag 50 mg/day, vonoprazan 20 mg/day, and prednisolone 10 mg/day.

Upon admission, his height and body weight were 175.6 cm and 61.4 kg, respectively, and his body mass index was 19.9 kg/m^2^. His blood pressure was 149/88 mmHg, heart rate was 81 beats per minute, and SpO_2_ was 98% on room air. In addition, his respiratory and heart sounds were normal. He had scars of shingles on both lower backs and presented neither edema nor purpura in both lower legs.

The patient’s laboratory findings upon admission are presented in Table 1. His platelets were 16,000/μL, and activated partial thromboplastin time (APTT) was 61.2 sec in admission. He presented with significant renal dysfunction, indicated by a urea nitrogen level of 53.6 mg/dL, a creatinine level of 4.36 mg/dL, mild hematuria, mild proteinuria (0.6 g/g Cr spot urine), elevated β2-microglobulin at 7600.7 μg/L, and other immunological abnormalities, including positive antinuclear antibodies (1:40), anti-dsDNA antibodies 63.4 U/mL, anti-ssDNA antibodies 251.7 U/mL, and lupus anticoagulant 2.1. His anti-neutrophil cytoplasmic antibody (ANCA) and anti-glomerular basement membrane (GBM) antibody results were negative. ADAMTS13 activity was 86％, and ADAMTS13 inhibitor was negative.

**Table 1:** Laboratory findings RBC, red blood cell; Hb, hemoglobin; WBC, white blood cell; PT-INR, prothrombin time-international normalization ratio; APTT, activated partial thromboplastin time; CRP, c-reactive protein; AST, aspartate aminotransferase; ALT, aspartate aminotransferase; BUN, blood urea nitrogen; eGFR, estimated glomerular filtration rate; HbA1c, hemoglobin A1c; Ig-, immunoglobulin; MPO-ANCA, myeloperoxidase-anti-neutrophil cytoplasmic antibody; PR3-ANCA, proteinase3 anti-neutrophil cytoplasmic antibody; GBM, glomerular basement membrane; β2GP-I, beta2 glycoprotein-I; ADAMTS-13, a disintegrin and metalloproteinase with thrombospondin motifs 13; β2-MG, beta2-microglobulin; NAG, N-acetyl-β-glucosaminidase

Parameters	Values	Reference Range
RBC	3.07×10^６^/μL	4.25-5.55×10^6^/µL
Hb	9.3 g/dL	13.7-16.8 g/dL
Hematocrit	28.0%	40.7-50.1％
WBC	8900/µL	3.3-8.6×10^3^/µL
Neutrophil	76.0％	40.0-60.0％
Eosinophil	3.0％	0.0-7.0％
Basophil	0.0％	0.0-3.0％
Lymphocyte	11.0％	25.0-45.0％
Monocyte	10.0％	0.0-8.0％
Platelet	16×10^3 ^/μL	158-348×10^3^/µL
PT-INR	1.06	0.8-1.1
APTT	61.2 sec	25.0-37.9 sec
CRP	4.99 mg/dL	0.00-0.14 mg/dL
Na	137 mmol/L	138-145 mmol/L
K	4.1 mmol/L	3.6-4.8 mmol/L
Cl	105 mmol/L	101-108 mmol/L
Calcium	8.9 mg/dL	8.8-10.1 mg/dL
Phosphate	3.2 mg/dL	2.7-4.6 mg/dL
Total protein	6.9 g/dL	6.6-8.1 g/dL
Albumin	3.5 g/dL	4.1-5.1 g/dL
AST	11 U/L	13-30 U/L
ALT	15 U/L	10-42 U/L
T-bil	0.6 mg/dL	0.4-1.5 mg/dL
LDH	217 U/L	124-222 U/L
BUN	53.7 mg/dL	8.0-20.0 mg/dL
Cre	4.36 mg/dL	0.65-1.07 mg/dL
eGFR	12.3 mL/min/1.73m^2^	>60 mL/min/1.73m^2^
Glu	109 mg/dL	73-109 mg/dL
HbA1c	4.4％	4.9-6.0％
IgG	1309 mg/dL	861-1747 mg/dL
IgA	254 mg/dL	93-393 mg/dL
IgM	50 mg/dL	33-183 mg/dL
C3	118.8 mg/dL	73.0-138.0 mg/dL
C4	35.9 mg/dL	11.0-31.0 mg/dL
Haptoglobin	73.9 mg/dL	19.0-170.0 mg/dL
Antinuclear antibody	x40 (HOMO)	0-x40
Anti-dsDNA antibody	63.4 IU/mL	≦12.0 IU/mL
Anti-ssDNA antibody	251.7 IU/mL	0.1-7.0 U/L
Anti-Sm antibody	2.5 U/mL	≦20 U/mL
MPO-ANCA	<0.2 IU/mL	0.0-3.5 IU/mL
PR3-ANCA	<0.6 IU/mL	0.0-2.0 IU/mL
Anti-GBM antibody	<1.5 U/mL	0.0-7.0 U/mL
Anticardiolipin IgG antibody	＜4.0 U/mL	0-12 U/mL
Anti-β2GP-I antibody	<1.3 U/mL	0.0-3.5 U/mL
Lupus anticoagulant	2.1 (positive)	0.0-1.2
ADAMTS13 activity	0.86 IU/mL (86%)	0.10- IU/mL (10％)
ADAMTS13 inhibitor	<0.5 BU/mL	0.5 BU/mL
Urinary pro/Urinary Cr	0.6 g/gCr	0.0-0.15 g/gCr
Urinary sediment	-	-
Red blood cells	>100/HPF	≦4/HPF
White blood cells	5-9/HPF	≦4/HPF
Urinary β2-MG	7600.7 μg/L	0.0-250 µg/L
Urinary NAG	13.6 U/L	0.0-11.5 U/L
Urinary M protein	Negative	Negative
Electrophoresis of protein	Negative	Negative

We suspected rapid progressive glomerulonephritis (RPGN) due to the rapid onset of renal dysfunction along with visible hematuria. Additionally, lupus nephritis is indeed a possibility in this case due to the presence of positive antinuclear antibodies and anti-dsDNA antibodies. We detected prolonged APTT and elevating lupus anticoagulant (LA), but there were no thrombotic events. We could not definitively diagnose antiphospholipid antibody syndrome (APS) with only a single instance of LA positivity, but it remained a concern. We considered the possibility of active lupus nephritis, and he was administered 500 mg of methylprednisolone for three days. A renal biopsy was performed on the hospital day 18.

Renal biopsy findings

**Figure 2:**
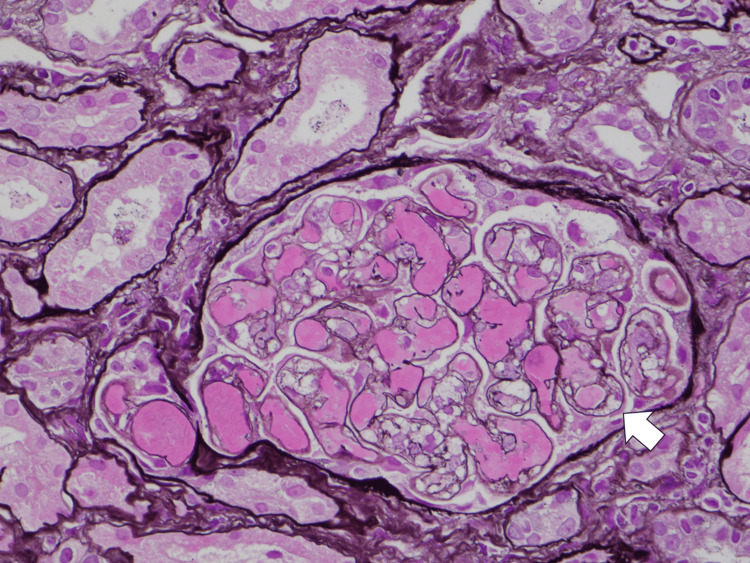
Light microscopic finding of periodic acid-methenamine silver staining x200 Diffuse double contour of the glomerular basement membrane and fibrin thrombi are observed in glomerular capillary loops. The arrow indicates the double contour.

**Figure 3:**
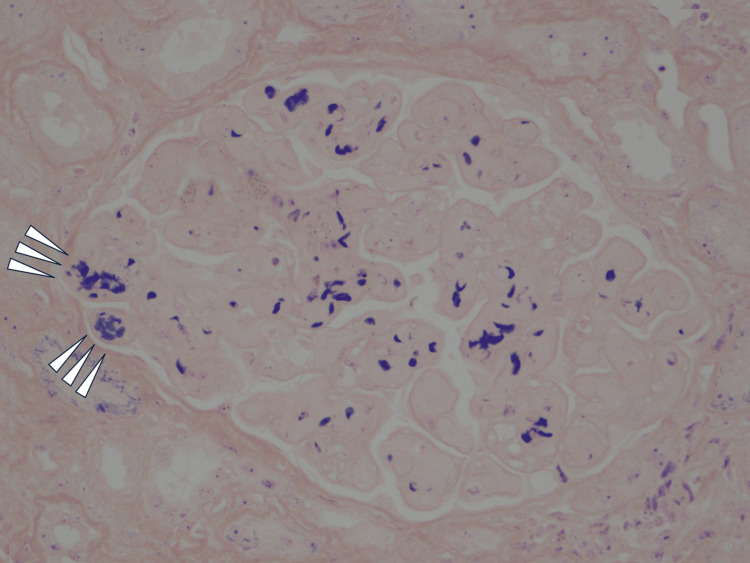
Phosphotungstic acid hematoxylin staining (x400) Arrow heads indicate that there are fibrin thrombi in the capillary loop.

**Figure 4:**
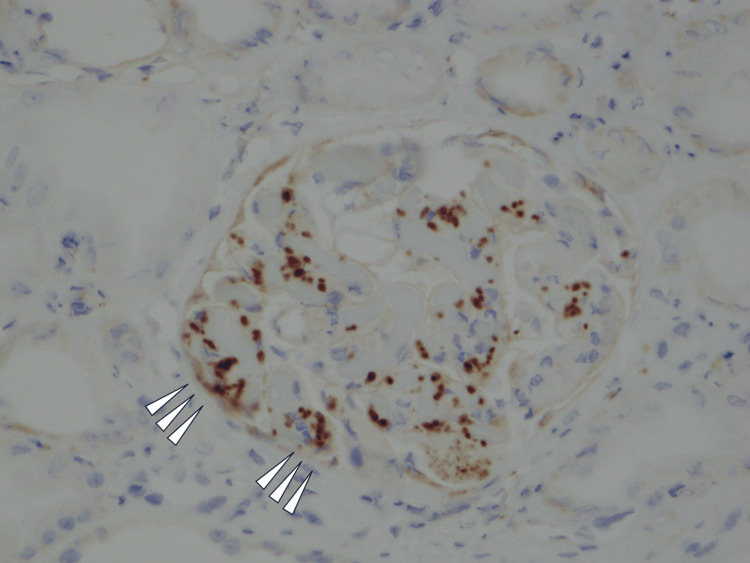
CD-61 staining (x200) Arrow heads indicate the existence of platelets in the thrombi.

**Figure 5:**
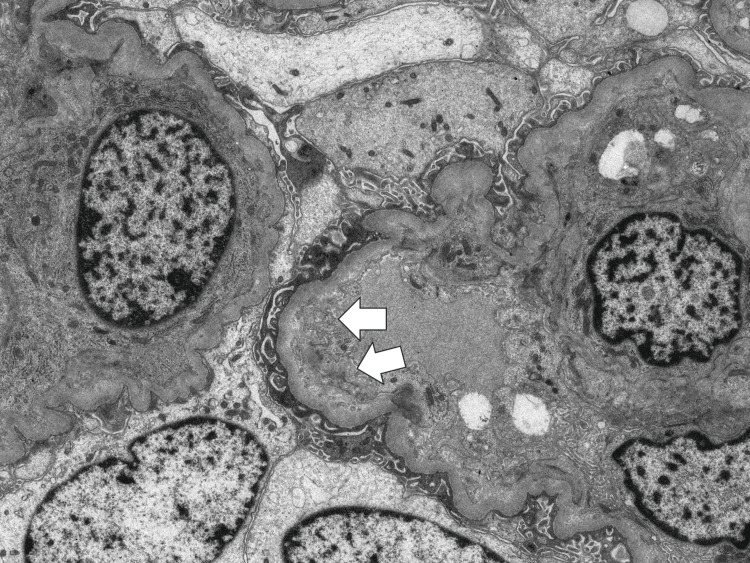
Electron microscopic staining (x1500) Diffuse foot process loss and subendothelial swelling were observed. Arrows show subendothelial swelling.

The cortex-to-medulla ratio was 6:4. There were 16 glomeruli: one was global sclerosis, and three were highly collapsed. The remaining 12 glomeruli showed global subendothelial expansion with endocapillary hypercellularity, double contour in the glomerular basement membrane, and congestion (Figure 2). Only mild interstitial fibrosis and tubular atrophy were observed. Arterioles showed onion skin lesions, subendothelial swelling, and enlarged endothelial cells, which suggested endothelial disorders. Congestive glomeruli showed phosphotungstic acid-hematoxylin-positive fibrin deposits (Figure 3) and CD61-positive platelets (Figure 4), which suggested platelet thrombus. All immunofluorescence was negative. Electron microscopy showed no electron-dense deposits but a loss of foot processes and endothelial swelling (Figure 5). Immunofluorescence did not show significant depositions; therefore, lupus nephropathy was ruled out. He was diagnosed with renal limited thrombotic microangiopathy (TMA) at this time due to endothelial disorders observed in the kidney.

Clinical course

We examined the etiologies of TMA; there was no coexistence of infection, hypocomplementemia, decline of ADAMTS13 activity, the appearance of ADAMTS13 inhibitor, or decline of haptoglobin as mentioned above, suggesting that this case was unlikely to have hemolytic uremic syndrome (HUS) and thrombotic thrombocytopenic purpura. Although autoantibodies were weak positive and anti-ds-DNA antibody positive, TMA was thought not to be associated with systemic lupus erythematosus due to the absence of significant deposition in immunofluorescence.

Eltrombopag-induced renal-limited TMA was suspected due to rapid renal dysfunction after increasing eltrombopag from 12.5 mg/day to 25 mg/day. Hence, weekly romiplostim was started instead of eltrombopag. Furthermore, we switched to 40 mg/day of prednisolone after the administration of 500 mg of methylprednisolone. However, renal function remained at Cre 4.3 to 4.5 mg/dL, and the estimated glomerular filtration rate (eGFR) was 11 to 13 mL/min/1.73m^2^. We concluded that the recovery of renal function was impossible and tapered off prednisolone for renal dysfunction.

In February 2023, he had acute myocardial infarction (AMI) and acute heart failure. He underwent percutaneous coronary intervention (PCI) due to 99% stenosis in the #13 segment of his left circumflex artery. His renal function worsened following the use of contrast medium. Due to heart failure with preserved ejection fraction, he developed pulmonary edema. We explained to him the necessity of renal replacement therapy. Although he wanted to select peritoneal dialysis, it was difficult for him to control excess fluid volume because of decreasing cardiac function. He finally agreed to initiate hemodialysis, and we planned to have surgery for arteriovenous fistula (AVF) in April 2023. However, he gained weight in March 2023, and many diuretics - furosemide 240 mg/day, azosemide 60 mg/day, torasemide 8 mg/day, and tolvaptan 15 mg/day - could not attenuate his pulmonary congestion. As echocardiography showed aggravation of mitral insufficiency and tricuspid insufficiency, we expected he had a high risk of developing acute heart failure after AVF surgery. Therefore, hemodialysis was initiated after a double-lumen catheter was inserted in early April 2023. Removing excess water, AVF surgery was undergone on his left arm eight days after the catheter insertion. No heart failure symptoms were observed after surgery, and he was discharged home after confirming his AVF could be cannulated two weeks after the surgery. When he went to the hospital for maintenance dialysis in early May 2023, thrombotic obstruction above anastomotic AVF was found, and he was required to receive vascular access intervention therapy.

As he had multiple histories of thrombosis, AMI, and AVF obstruction, we reassessed lupus anticoagulant, and it turned out to be positive in November 2021. In this case, LA was positive in May 2022. We diagnosed APS because he had several histories of thrombosis, and LA was positive two times at 12 weeks apart. As he took 100 mg of aspirin after the AMI, we did not add other antiplatelets or anticoagulants for APS.

The patient was subsequently transferred to a nearby dialysis center and receives outpatient maintenance dialysis three times a week. The dialysis shunt was frequently stenotic and occluded by a thrombus, leading to its reconstruction in November 2023. Since then, there has been no evidence of shunt occlusion. Romiplostim is administered once a week, and the platelet count remains between 10,000 and 300,000/μL.

## Discussion

In this case, renal biopsy indicated endothelial disorders, and TMA was suggested. We examined the cause of TMA, but no infections, hypocomplementemia, or reduced ADAMTS13 activity were found. After increasing eltrombopag, a decline in renal function was observed, leading us to suspect drug-induced TMA. Eltrombopag is a thrombopoietin-receptor agonist that promotes the proliferation and differentiation of cells in the megakaryocytic lineage. Eltrombopag can activate platelets easily [3] and be a risk for thrombosis, especially in cases with thrombotic predisposition, a history of splenectomy, and coexisting APS [4].

We suspected eltrombopag-induced TMA, whereas this case satisfied the classification criteria for APS. The classification criteria for definite APS need at least one of the clinical criteria: (1) Vascular thrombosis, (2) Pregnancy morbidity; and laboratory criteria: (1) LA is positive more than two at least 12 weeks apart, (2) Anticardiolipin (aCL) antibody of IgG and/or IgM isotype are positive in medium or high titer on more than two at least 12 weeks apart, (3) Anti-β2 glycoprotein-I antibody of IgG and/or IgM isotype are positive in medium or high titer on more than two at least 12 weeks apart [5].

There are not so many cases of eltrombopag-induced acute kidney injury, but eight cases have been reported [6-13]. In Table 2, cases, where kidney biopsy was performed, showed pathology of TMA except in two cases, which suggested effects of thrombosis caused by eltrombopag. The time to onset varied among cases, ranging from as short as five days to as long as approximately six weeks. In previous reports, acute kidney injury typically occurred after the initiation of eltrombopag. However, in this case, the patient's renal dysfunction was associated with the escalation of eltrombopag dosage. At baseline, the patient's creatinine level was 0.7 mg/dL, and the eGFR ranged from 90 to 100 mL/min/1.73 m². Four weeks after the eltrombopag dose was increased from 12.5 mg to 25 mg in April 2022, his creatinine rose to 1.3 mg/dL, and eGFR declined to 45 mL/min/1.73 m². The renal function further deteriorated following another dose increase to 37.5 mg in August 2022, with his serum creatinine reaching 4.36 mg/dL and eGFR dropping to 12.3 mL/min/1.73 m². There was a report of deep venous thrombosis in a patient with ITP coexisting APS after eltrombopag was administered [14]. In this point, APS also played a role in developing TMA; however, renal dysfunction in this case developed shortly after augmenting eltrombopag, suggesting that eltrombopag could trigger TMA.

**Table 2:** Literature review and this case ITP, idiopathic thrombotic purpura; SLE, systemic lupus erythematosus; TMA, thrombotic microangiopathy; APS, antiphospholipid syndrome; FSGS, focal segmental glomerulosclerosis

No.	Age	Sex	Background diseases	Treatment	Pathology in Kidney Biopsy	Outcome	Reference
１	54	Male	ITP， APS	Only discontinuing drugs	Did not perform	No availability	[6]
2	19	Female	ITP， APS	Steroid, Plasma exchange, Rituximab	TMA	Improve renal function	[7]
3	46	Female	ITP	Splenectomy, hemodialysis	FSGS, collapsing variant	Improve renal function	[8]
4	77	Male	ITP	Steroid	FSGS	Improve renal function	[9]
5	80	Male	ITP， APS	Steroid, Plasma exchange	TMA	Improve renal function	[10]
6	64	Female	ITP	Steroid, Plasma exchange	TMA	Improve renal function	[11]
7	45	Male	ITP	Steroid, hemodialysis	Did not perform	Improve renal function	[12]
8	60	Female	ITP， APS	Plasma exchange	Thrombosis	Improve renal function	[13]
This case							

General treatments of drug-induced TMA are mainly decreasing or discontinuing suspected drugs and supportive therapy. However, many cases cannot improve their renal function, only discontinue drugs; some patients were treated with plasma exchange or rituximab [6]. Plasma exchange was performed for the mechanical removal of autoantibodies. In cases of eltrombopag-induced TMA, some cases improved with discontinuing drugs alone, and some cases required prednisolone or plasma exchange.

This case did not improve after discontinuing eltrombopag and administrating prednisolone, so we considered initiating plasma exchange. However, plasma exchange was not performed because the number of cases in which plasma exchange was effective was small, and the level of evidence was not regarded as high. The efficacy of plasma exchange for eltrombopag-induced TMA remains unclear, and further studies are required to elucidate the mechanisms of eltrombopag-induced TMA. Eventually, his renal function could not improve after he was referred to our department and required to initiate renal replacement therapy.

At first, we diagnosed kidney-limited TMA because systemic thrombosis had not been shown except for renal pathology. Although AMI and AVF thrombosis may be attributed to APS, TMA could be essential in developing vascular events. Some cases of deep venous thrombosis following eltrombopag were reported in patients with APS. Therefore, further thrombosis is possible, and cautious follow-up is needed. Additionally, there remained a possibility of atypical HUS, as genetic testing was not performed.

## Conclusions

We described a case of eltrombopag-induced TMA complicated by ITP and initiated hemodialysis. As eltrombopag is used for ITP and may induce the development of thrombosis, more careful follow-up is required as the risk of thrombosis increases, especially in cases with APS.
